# Supplementary material for: Novel mutation SLFN14 T853fs associated with inherited macrothrombocytopenia
Source: Mol Ther Nucleic Acids. 2025 May 7;36(2):102554. doi: 10.1016/j.omtn.2025.102554 (PMC12159219; doi:10.1016/j.omtn.2025.102554)
Supplement: Document S1. Figure S1 [file mmc1.pdf]

OMTN, Volume *36*

Supplemental information

Novel mutation *SLFN14* T853fs associated with inherited macrothrombocytopenia

Haixiao Xie, Shiyi Tang, Jianmin Shao, Ming Yang, Huida Tong, Linhua Zhang, Mingzhu Zhong, Xiaomin Yu, Laixi Bi, Yuming Wang, Rongying Ou, Chen Ling, and Liqing Zhu

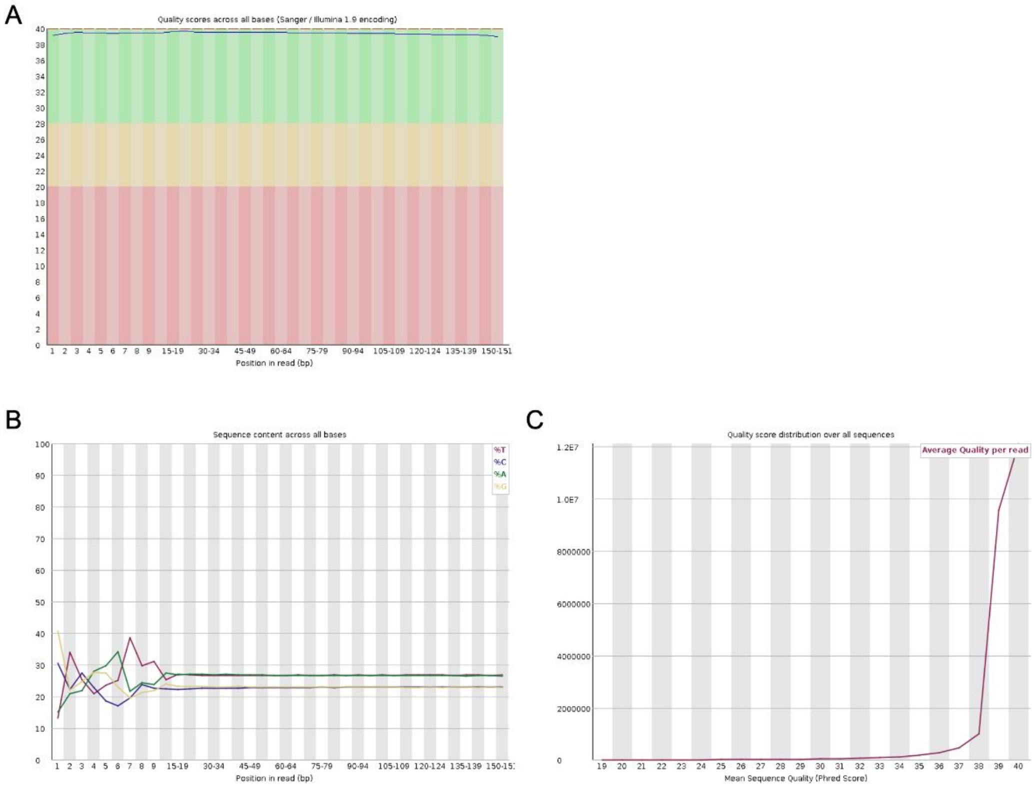

Figure S1. Representive quality control analysis of RNA-seq. (A) Per-base sequence quality scores (y-axis) across read positions (x-axis in bp). Colors (green [high] to red [low]) indicate quality variations. **(B)** Per-base sequence composition. Nucleotide content percentage (y-axis) across read positions (x-axis in bp). Lines represent %T (red), %C (blue), %A (green) and %G (yellow). **(C)** Read quality distribution. Y-axis: read count. X-axis: mean sequence quality.
