# Supplementary material for: Novel mutation SLFN14 T853fs associated with inherited macrothrombocytopenia
Source: Mol Ther Nucleic Acids. 2025 May 7;36(2):102554. doi: 10.1016/j.omtn.2025.102554 (PMC12159219; doi:10.1016/j.omtn.2025.102554)
Supplement: Document S2. Article plus supplemental information [file mmc2.pdf]

Molecular Therapy
Nucleic Acids
Original Article

Novel mutation *SLFN14* T853fs associated with inherited macrothrombocytopenia

Haixiao Xie,[1,9] Shiyi Tang,[1,2,9] Jianmin Shao,[3,9] Ming Yang,[4,9] Huida Tong,[1] Linhua Zhang,[5] Mingzhu Zhong,[6] Xiaomin Yu,[1] Laixi Bi,[7] Yuming Wang,[1] Rongying Ou,[6] Chen Ling,[1,4] and Liqing Zhu[8]

[1]Department of Clinical Laboratory, the First Affiliated Hospital of Wenzhou Medical University, Wenzhou, Zhejiang 325000, China; [2]Department of Blood Transfusion, Zhuzhou Center Hospital, Zhuzhou, Hunan 412000, China; [3]Department of Clinical Laboratory, Yongjia Hospital of TCM, Wenzhou, Zhejiang 325100, China; [4]State Key Laboratory of Genetics and Development of Complex Phenotypes and Engineering Research Center of Gene Technology (Ministry of Education), School of Life Sciences, Zhongshan Hospital, Fudan University, Shanghai 200438, China; [5]Department of Clinical Laboratory, the People's Hospital of Yuhuan, Taizhou, Zhejiang 317600, China; [6]Department of Obstetrics and Gynecology, the First Affiliated Hospital of Wenzhou Medical University, Wenzhou, Zhejiang 325000, China; [7]Department of Hematopathology, the First Affiliated Hospital of Wenzhou Medical University, Wenzhou, Zhejiang 325000, China; [8]Department of Clinical Laboratory, Peking University Cancer Hospital & Institute, Beijing 100142, China

***SLFN14*-related inherited thrombocytopenia (*SLFN14*-related IT) is a hereditary disorder involving ribosomopathy and platelet dysfunction. Affected patients exhibit significant bleeding tendencies. To date, five affected pedigrees have been reported, all harboring mutations within the "ATPase associated with diverse cellular activities" (AAA) domain. In this study, we identified a novel T853fs variant located in the "helicase" domain. SLFN14 expression was markedly reduced in platelets from the patients and in Meg-01 cells transfected with T853fs plasmid. Functional assays revealed a defection of T853fs variant in both arachidonic acid (AA)-induced aggregation and fibrinogen-induced adhesion. Unlike previously reported mutations in the AAA domain, which significantly upregulate ribosomal protein genes and mitochondrial translation pathways, the T853fs mutation identified in this study did not affect mitochondrial translation. Immunofluorescence assay showed that T853fs variant exhibited diffuse cytoplasmic localization. Further RNA sequencing (RNA-seq) analysis revealed the significant regulation of T853fs mutation on pathways related to ion channels and dense granule, which are crucial to platelet function. In conclusion, this study identifies a new *SLFN14* mutation and highlights the phenotypic diversity of SLFN14-RT.**

INTRODUCTION

SLFN14, a member of the Schlafen protein family, was recently identified as being associated with inherited thrombocytopenia (IT), a disorder characterized by low platelet counts, impaired proplatelet elongation, reduced adenosine triphosphate (ATP) secretion, and increased bleeding tendencies. In the UK Genotyping and Phenotyping of Platelets study, four dominant missense mutations in the *SLFN14* gene were identified in 12 patients from three unrelated families.[1] Another missense mutation was reported later.[2] *SLFN14* exhibits endoribonuclease activity targeting aberrant RNA and plays a role in the degradation of ribosomes, tRNAs, and mRNAs during platelet maturation.[3] As a result, IT associated with *SLFN14* mutations is accompanied by increased degradation of ribosome-related RNA in platelets and megakaryocytes.[4]

Recent biochemical and structural studies of other SLFN family members have gradually revealed their molecular characteristics.[5–8] The N-terminal region has been identified as the key domain for endoribonuclease activity. The M-domain, which connects the N-terminal and C-terminal regions, contains a specific SWAVDL motif (Ser-Trp-Ala-Asp-Leu) and serves as a potential protein-interaction region. The C-terminal domain features extensive COOH-terminal extensions and houses RNA/DNA helicases. These helicases participate in DNA replication, translation, ribosome biogenesis, and DNA repair through ATP hydrolysis-driven remodeling of RNA/DNA strands.[9,10] All known *SLFN14* mutations (K218E, K219E/N, V220D, and R223W) are located within the "ATPase associated with diverse cellular activities" (AAA) protein domain. These mutant proteins exhibit a tendency to oligomerize, which may explain the dominant-negative effect observed in heterozygous mutations affecting patients' platelets.[1,11]

The relationship between *SLFN14* mutation and thrombocytopenia remains unclear. *SLFN14* variants, when were transfected into cells, showed a reduction in expression of over 50%, likely due to partial misfolding and subsequent post-translational degradation. Notably,

Received 19 October 2024; accepted 30 April 2025;
https://doi.org/10.1016/j.omtn.2025.102554.

[9]These authors contributed equally

Correspondence: Rongying Ou, Department of Obstetrics and Gynecology, the First Affiliated Hospital of Wenzhou Medical University, Wenzhou, Zhejiang 325000, China.
E-mail: ourongying@163.com
Correspondence: Chen Ling, Department of Clinical Laboratory, the First Affiliated Hospital of Wenzhou Medical University, Wenzhou, Zhejiang 325000, China.
E-mail: lingchenchina@fudan.edu.cn
Correspondence: Liqing Zhu, Department of Clinical Laboratory, Peking University Cancer Hospital & Institute, Beijing 100142, China.
E-mail: wzzhuliqing@126.com

Published by Elsevier Inc. on behalf of The American Society of Gene and Cell Therapy.
This is an open access article under the CC BY license (http://creativecommons.org/licenses/by/4.0/).

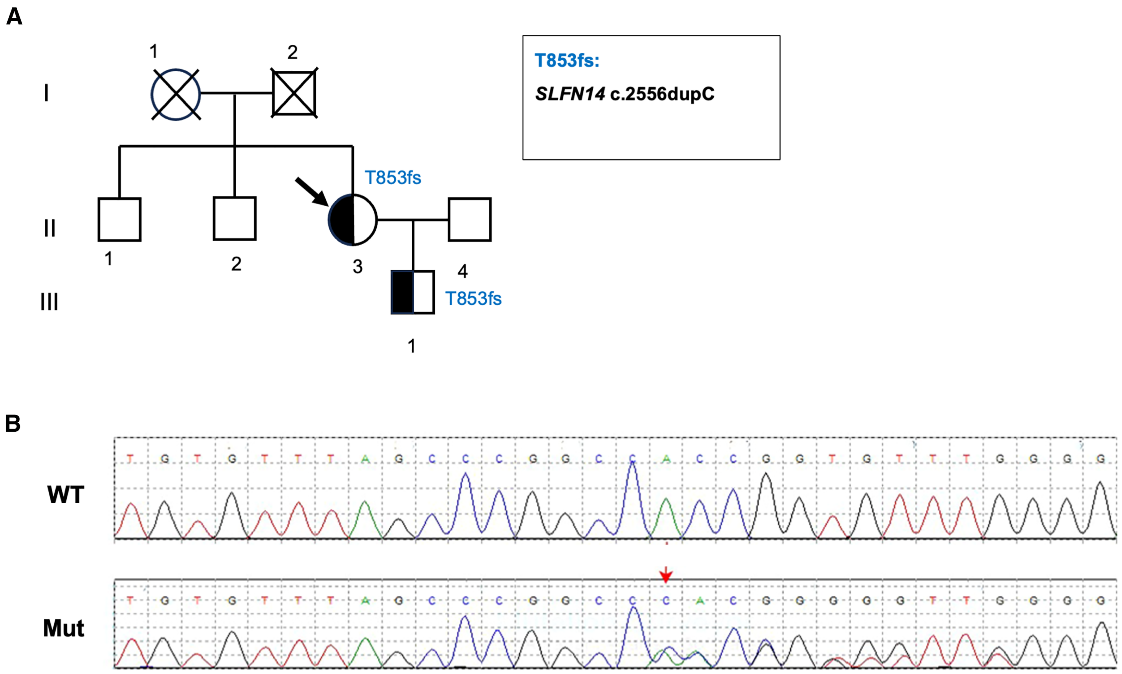

Figure 1. Novel T853fs mutation of *SLFN14* associated with inherited macrothrombocytopenia

(A) Pedigree showing affected family members (half black symbols) with IT. The presence of the Thr853fs *SLFN14* variant was identified through WES, and is indicated when tested. The black arrow indicates the proband. (B) Representative Sanger sequencing electropherogram of wild-type (top) and mutated (bottom) *SLFN14* are shown. The red arrow indicates the position of c.2556dupC.

all variants co-localized with ribosomes and demonstrated rRNA endonucleolytic degradation activity.[12] Interestingly, heterozygous K208N mice (the equivalent of human K219N) exhibited microcytic erythrocytosis, hemolytic anemia, splenomegaly, and abnormal thrombus formation, with unaffected platelet count, function, and morphology.[13] This mutation, observed in both humans and mice, leads to defects in thrombopoiesis and erythropoiesis, respectively. These findings highlight that the precise mechanistic role of *SLFN14* in blood cell development remains unknown.

In this study, we identified a novel T853fs mutation in a *SLFN14*-related inherited thrombocytopenia pedigree. Unlike the previously reported five mutations (K218E, K219E/N, V220D, and R223W), which are located in the N-terminal domain, this is the first mutation found in the C-terminal "helicase" domain of *SLFN14*. We investigated the structural characteristics and platelet properties associated with the T853fs mutation, and further explored the underlying pathways involved in macrothrombocytopenia. This study expands our understanding of *SLFN14*-related IT by supplementing existing knowledge of mutations and mechanisms.

RESULT

Novel T853fs mutation of *SLFN14* associated with inherited macrothrombocytopenia

In this study, a pedigree with inherited macrothrombocytopenia were identified. The proband (patient II:3), a 34-year-old woman, had a low platelet count of 34×10^9/L (normal range: 125–350 $\times 10^9$/L) when recruited to the study. She complained of a history of heavy menstrual bleeding, nasal mucosal bleeding, and postpartum hemorrhage, with a bleeding score of 9 according to the ISTH Bleeding Assessment Tool. Her son (patient III:1) also had a low platelet count of 42×10^9/L, accompanied with nasal cavity bleeding and excessive bleeding after tooth extraction (Figure 1A).

Whole exome sequencing (WES) analysis of the proband identified a novel heterozygous c.2556dupC mutation in the *SLFN14* gene (Figure 1B), which resulted in a Thr853 frameshift in exon 4 (T853fs). This mutation was also confirmed positive in the proband's son through Sanger sequencing, who was likewise suffering from thrombocytopenia. In contrast, it was negative in other family members with normal platelets count.

Structure characters of *SLFN14* T853fs variant

To date, five variants (K218E, K219E/N, V220D, and R223W) of *SLFN14* related to thrombocytopenia have been reported, all focusing on the hotspot within the AAA protein domain. In contrast, the novel c.2556dupC mutation discovered in this study was located in helicase domain of C-terminal, which was only possessed by *SLFN* group III, including *SLFN5*, *SLFN11*, *SLFN13*, and *SLFN14*.[6]

Three-dimensional (3D) modeling using Pymol predicted that the c.2556dupC mutation would lead to a deletion of 27 arachidonic

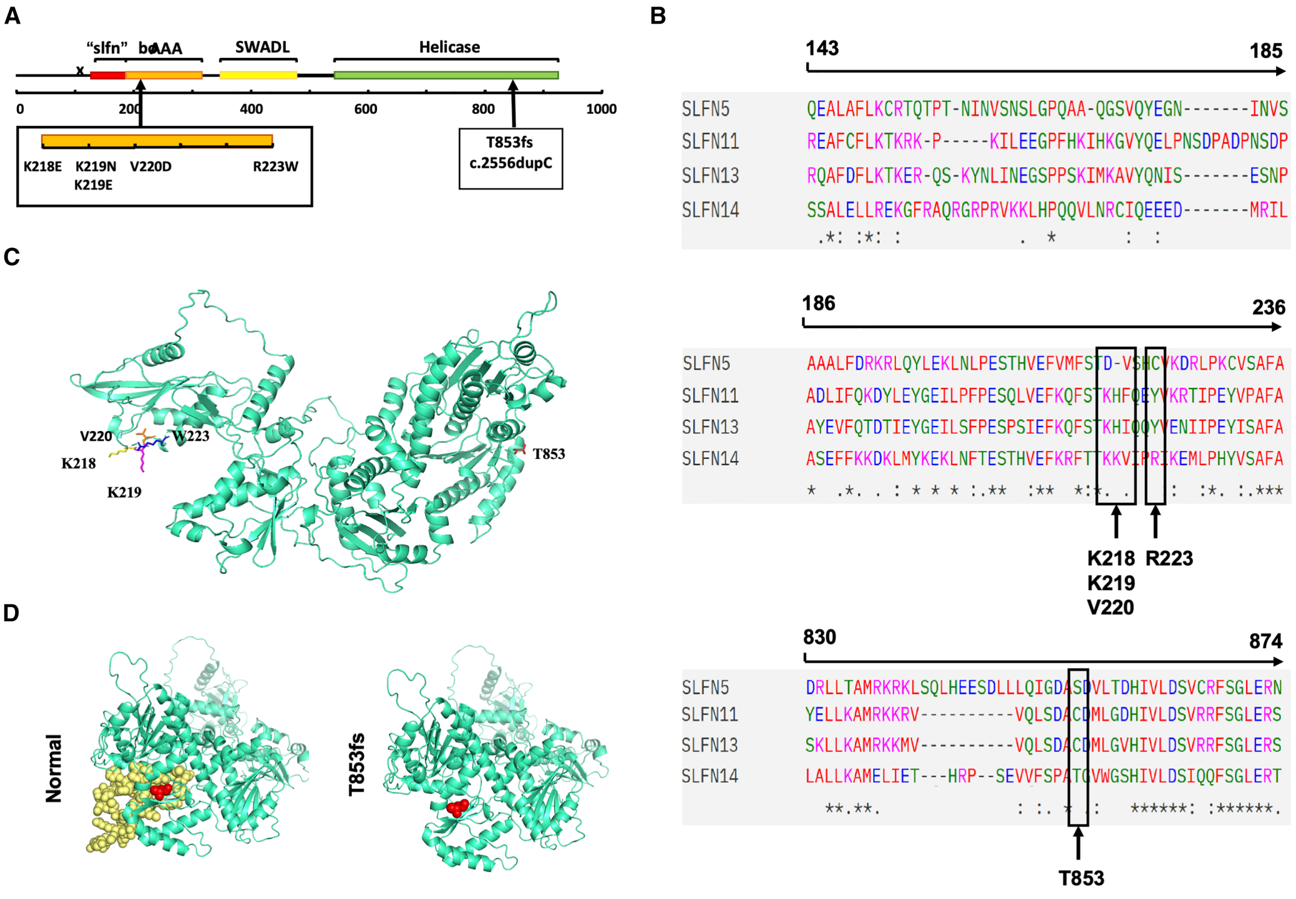

Figure 2. Structure characters of *SLFN14* T853fs variant

(A) Linear schematic diagram of the *SLFN14* structural domains at the DNA level. (B) Conservation of the protein sequence among SLFN family. "*" indicates a fully conserved residue; ":" a conservation between groups of strongly similar properties, scoring >0.5 in the Gonnet PAM 250 matrix; and "." a conservation between groups of weakly similar properties, scoring ≤0.5 in the Gonnet PAM 250 matrix. Loci in previously reported literatures are indicated. (C) Overview of structure of the complete *SLFN14* monomer. Mutation loci have been indicated. (D) The comparison of the predicted structure of WT and T853fs *SLFN14*. The T853fs mutation resulted in a truncated *SLFN14*.

acid (AA) in exon 4, resulting in a truncated *SLFN14* (Figures 2A–2C). Sequence analysis showed the homology of T853 is relatively low (Figure 2D). Pathogenicity prediction using Ensembl variants database and VEP software both classified the T853fs variant as "high" risk, suggesting it is pathogenic.

T853fs leads to low expression level of SLFN14

Since Fletcher et al. reported that several *SLFN14* mutations lead to exhibited decreased protein expression without affecting transcription levels,[12] we constructed the wild-type (WT) and T853fs mutant *SLFN14* overexpression plasmids. We then transfected them to HEK293T and Meg-01 cells individually. Real time qPCR showed that endogenous *SLFN14* expression in both HEK293T and Meg-01 cells were undetectable. Interestingly, the *SLFN14* mRNA expression from T853fs plasmid was significantly reduced compared to the WT (Figure 3A), which was further confirmed by western blot analysis, showing a 50%–80% reduction (Figure 3B). Platelet lysates from the proband revealed a nearly 40% decrease of *SLFN14* with heterozygous T853fs mutation compared to controls (Figure 3C), verified the truncated expression of variant *SLFN14*.

SLFN14 has been identified as a ribosome-associated endoribonuclease in rabbit reticulocytes, regulating the cleavage of rRNA and ribosome-related mRNA in the presence of Mg^{2+}.[3] The rRNA degradation of SLFN14-WT and SLFN14-T853fs overexpressed HEK293T cells were examined. However, no obvious bands of degraded rRNA were observed in platelets carrying T853fs mutation. Thus, the novel T853fs mutation in *SLFN14* had no effect on ribosome degradation activity (Figure 3D). Additionally, CCK-8 assays conducted on Meg-01 cells transfected with EV, SLFN14-WT, or SLFN14-T853fs plasmids showed no significant differences in viable cell number (Figure 3E), suggesting that the T853fs variant does not affect Meg-01 cell proliferation.

To investigate whether the T853fs mutation affects the subcellular location of SLFN14 protein, immunofluorescence assays were performed on SLFN14-WT and SLFN14-T853fs overexpressed

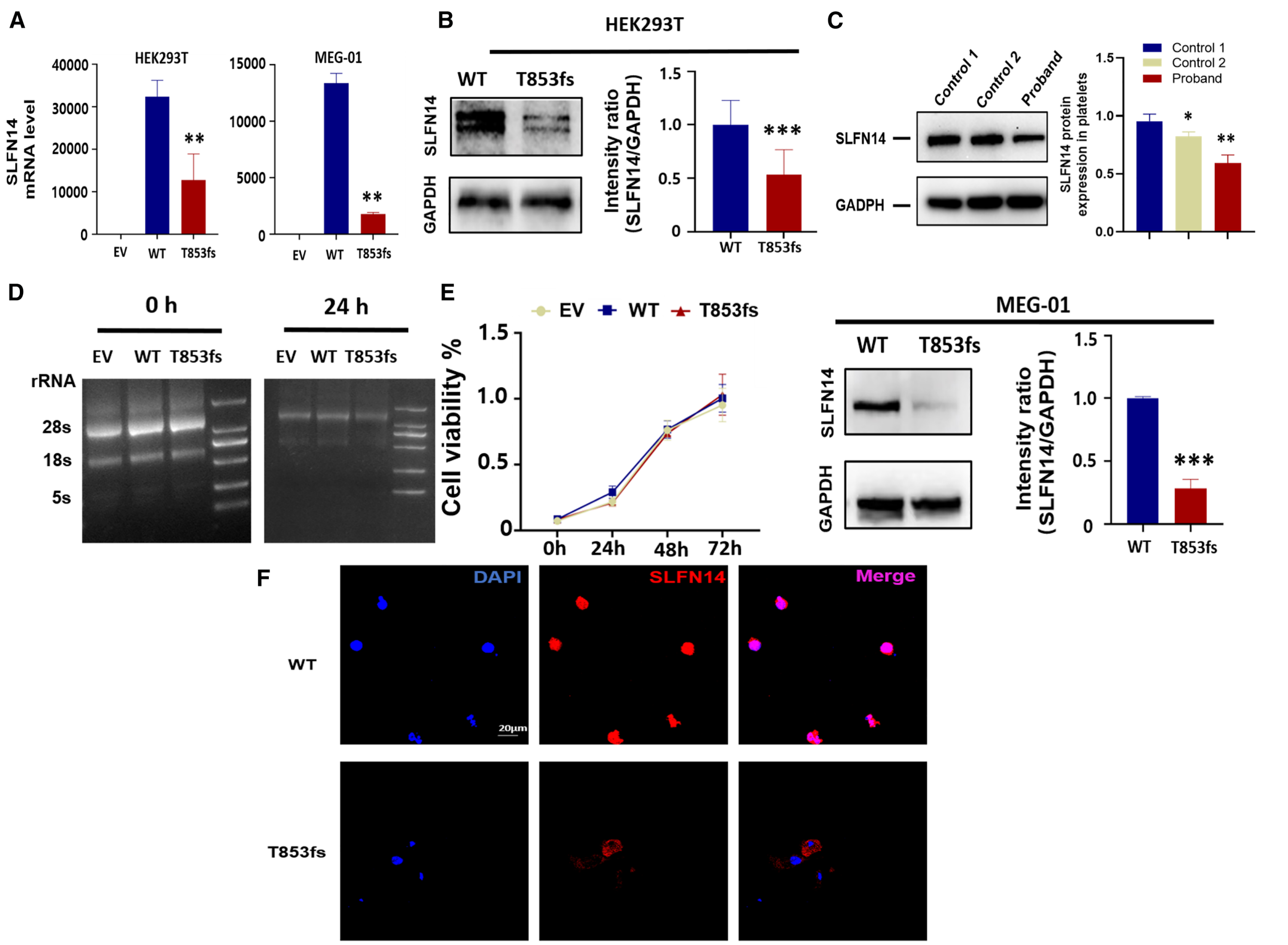

Figure 3. T853fs leads to low expression level of SLFN14

(A and B) HEK293T and Meg-01 cells were transfected with SLFN14-WT or SLFN14-T853fs overexpression plasmids for 48 h. The expressions of exogenous *SLFN14* were determined by (A) real time qPCR and (B) western blot. Both representative figures and quantification data for western blot are shown. GAPDH was used as an internal reference. (C) Western blot analysis of SLFN14 protein expression in platelet lysates of the proband and two healthy subjects (control 1 and control 2). Both representative figures and quantification data are shown. (D) rRNA degradation in *SLFN14*-WT and *SLFN14*-T853fs) overexpressing HEK293T cells was assessed by denaturing agarose/formaldehyde gel electrophoresis ($n = 3$ independent experiments). (E) Cell proliferation of MEG-01 cells that were transfected by empty vector (EV), SLFN14-WT, or SLFN14-T853fs overexpression plasmids was determined by Cell Counting Kit-8 (CCK-8) assay. (F) Immunofluorescence staining against SLFN14 (red) of MEG-01 cells transfected with SLFN14-WT or SLFN14-T853fs overexpression plasmids for 48 h. Nuclei were counterstained with DAPI (blue). Merged images indicate the overlay of SLFN14 and nuclei signals. Images were captured using a 60× objective. Scale bars: 20 μm. Data are presented as mean ± standard deviation (SD). Comparisons between two groups were conducted using unpaired two-tailed Student's t test. *$p < 0.05$, **$p < 0.01$, ***$p < 0.001$.

Meg-01 cells (Figure 3F). The results showed that the overexpressed WT SLFN14 was localized in the nucleus. However, the T853fs vairant was localized in cytoplasm.

T853fs variant leads to platelet defects

To further explore the association between T853fs variant and IT, peripheral blood from the proband was analyzed. Platelets were abnormally enlarged (Figure 4A), accompanied by increased MPV, platelet-large cell ratio (P-LCR%), and the proportion of immature platelets (Table 1). However, no significant abnormalities were observed in granulocytic or erythroid colonies.

Platelets isolated from the proband demonstrated lower spreading on fibrinogen coated coverslips (Figure 4B) and reduced aggregation in response to adenosine diphosphate (ADP) in comparison with normal controls, but their response to AA remained comparable to that of controls (Figure 4C).

Integrins GPIIb (CD41) and IIIa (CD61), function as the fibrinogen receptors on platelets, are highly expressed on platelets and their progenitor cells. They are considered as markers for platelet activation.[16] Flow cytometry showed a reduced expression of CD41 and CD61 on proband's platelets relative to normal controls

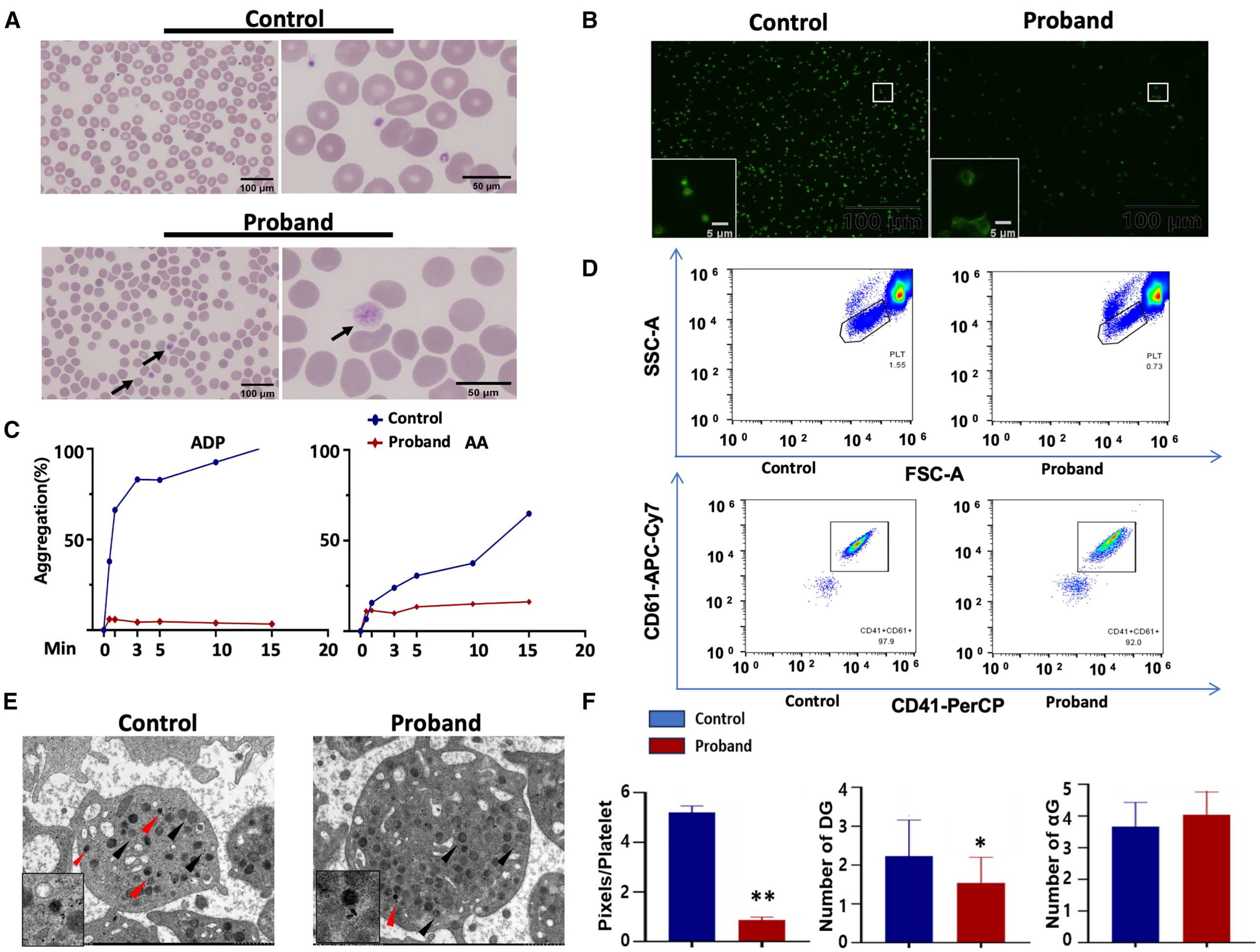

Figure 4. T853fs variant leads to platelet defects

(A) Peripheral blood smears (Giemsa stain) of patients II:3 showing platelet anisocytosis with the presence of small and large, hypogranular platelets. The arrows indicate enlarged platelets. Scale bars: 500 μm for the left panel and 50 μm for the right panel in (A). (B) Platelet spreading on 100 μg/mL fibrinogen coated coverslips. FITC-phalloidin was used to stain F-actin. Scale bar: 5 μm. (C) Representative impaired aggregation traces in platelet-rich plasma from the proband of the affected family. Diluted platelet-rich plasma was used to assess the percentage aggregation after stimulation with ADP (10 μM) and AA (1 mM). (D) Example plots of flow cytometry analysis of plasma from the proband, indicating the percentage of platelets and $CD41^+$/$CD61^+$ platelets. (E) Transmission electron microscopy imaging shows ultrastructural defects and decreased number of dense granules (red arrow) in platelets from the proband. The black arrow designates α-granules. Original magnification: ×8,000. (F) Quantified results of area per platelet (upper), dense granules (middle), and α-granules (lower). ADP, adenosine diphosphate; AA, arachidonic acid; DG, dense granules; αG, α-granules. Data are presented as mean ± SD. Comparisons between two groups were conducted using unpaired two-tailed Student's t test $*p < 0.05$, $**p < 0.01$.

(Figure 4D). This suggested that the T853fs variant exhibited impaired platelet adhesion and aggregation.

In line with these findings, electron microscopy identified a notable reduction in dense granules in platelets harboring the T853fs variant, while α granules showed no discernible differences compared to normal controls (Figures 4E and 4F).

Pathways involved in Thr853fs-associated inherited macrothrombocytopenia

Platelets are anucleate cells derived from megakaryocyte precursors, capable for *de novo* protein translation but lacking RNA transcription capacity.[17] The morphology and function of platelets are regulated by a series of genes during megakaryocyte differentiation. In this context, Meg-01 cells transfected with SLFN14-WT and SLFN14-T853fs were analyzed by RNA sequencing (RNA-seq) to explore the potential pathways involved in Thr853fs-associated inherited macrothrombocytopenia. The quality control analysis is shown in Figure S1.

Differential gene expression analysis yielded 37 upregulated and 51 downregulated genes (|log2 fold change| >1, adjusted p value < 0.05) (Figures 5A and 5B). Principal component analysis was performed for those differentially expressed genes (DEGs).

Table 1. Characteristics of platelets from patients with SLFN14-related thrombocytopenia

Genomic variant	Protein effect	Identity	Platelet count[a]: 125–350 × 10^9/L	Mean platelet volume (MPV) normal[b]: 6.8–13.5fL	Platelet aggregation defect (amplitude <50%) for the indicated agonist	Adhesion defect	rRNA fragments	Dense granules count
c.652 A>G	p. K218E	proband	89	13	**ADP (AA normal)**	NR	**increased**	NR
c.657 A>T	p. K219N	AI2	83	11.9	**ADP (AA normal)**	**tolerated**	**increased**	NR
		AII3	68	11.9	ADP	**tolerated**		**decreased**
		BII1	53	NR	NR	NR		NR
		BII3	49	NR	ADP	NR		NR
		BIII4	124	12.1	ADP	NR		decreased after activation
		BIV1	78	11.2	ADP	NR		NR
		BIV2	84	12.8	ADP	NR		decreased after activation
c.655 A>G	p. K219E	proband	76	NR	**ADP AA**	NR	NR	NR
c.659 T>A	p. V220D	III2	140	9.1	ADP	Tolerated	**increased**	NR
		III3	74	10.4	ADP	NR		NR
		IV2	110	9.3	ADP	NR		NR
		IV4	100	11.1	**ADP (AA normal)**	NR		**decreased**
		IV5	116	11.2	ADP	NR		NR
c.667 C>T	p. R223W	II1	87	12.1	NR	NR	**increased**	NR
		II2	91	21.0	NR	NR		NR
		III3	79	12.3	NR	NR		NR
c.2556dupC	p. T853fs	proband	34	13.2	**ADP, AA**	**impacted**	**normal**	**decreased**

The corresponding changes in the nucleotide sequence and their predicted effects on the resulting protein are shown.
[a]Platelet count <150 × 10^9 platelets/L is defined as thrombocytopenia.[14]
[b]The reference range of mean platelet volume (MPV, fL). The data of aggregation defect, adhesion defect, rRNA fragments, and dense granules count were obtained from published literature. NR: not reported in publication.[1,2,4,11–13,15]

The first principal component (PC1, 32.45%) and the second principal component (PC2, 28.97%) accounted for more than 50% of all variance between SLFN14-WT group and SLFN14-T853fs group (Figure 5C), suggesting the extensive impact of the SLFN14-T853fs mutation on the transcriptome. Consistent with the results of real time qPCR and western blot, the expression of *SLFN14* was significantly decreased (p = 0.023) in Meg-01 cells transfected with SLFN14-T853fs compared to Meg-01 cells transfected with SLFN14-WT (Figure 5D). Gene Ontology (GO) enrichment analysis of DEGs revealed that T853fs mutation significantly regulated multiple ion channel activity-related pathways, including calcium transmembrane transport, potassium channel regulation, and voltage-gated ion channel function. Notably, key ion channel genes exhibited marked expression changes. We validated the expression of those key ion channel genes using real time qPCR (Figure 5E), which showed significant downregulation of *CACNA1A*, a crucial gene related to calcium channel. Other regulated pathways included those related to ATPase, dense granule, and transmembrane transport (Figure S1). Unlike K219N mutation, which significantly upregulated ribosomal protein genes and pathways involved in mitochondrial translation, the T853fs mutation did not exert such effects. This was in line with our finding that the T853fs mutation in *SLFN14* has minimal effects on ribosome degradation activity.

DISCUSSION

SLFN14 is a recently identified gene associated with IT.[18] Unlike some IT cases, which are asymptomatic in platelet quality/funtion, patients with *SLFN14* mutations present with moderate thrombocytopenia, a history of severe bleeding, and platelet ATP secretion defects. To date, five heterozygous missense mutations (K218E, K219E/N, V220D, and R223W) have been reported, all located in the AAA domain, a region that is common among SLFN family members. The AAA domain contains key regions responsible for binding rRNA and tRNA,[19] which is involved in ATP binding, and plays a role in endoribonuclease activity.[20] Most patients with mutations in this domain exhibit similar phenotypes, including moderate thrombocytopenia, decreased ATP secretion, normal fibrinogen adhesion, reduced dense granules, a dominant inheritance pattern, and elevated rRNA fragments (Table 1). Studies confirmed that the K218E, K219N, and V220D variants in the AAA domain co-localize with ribosomes and mediate rRNA endonucleolytic degradation.[1] However, different mutations display varying degrees of heterogeneity. For example, K218E and K219N mutations significantly degrade

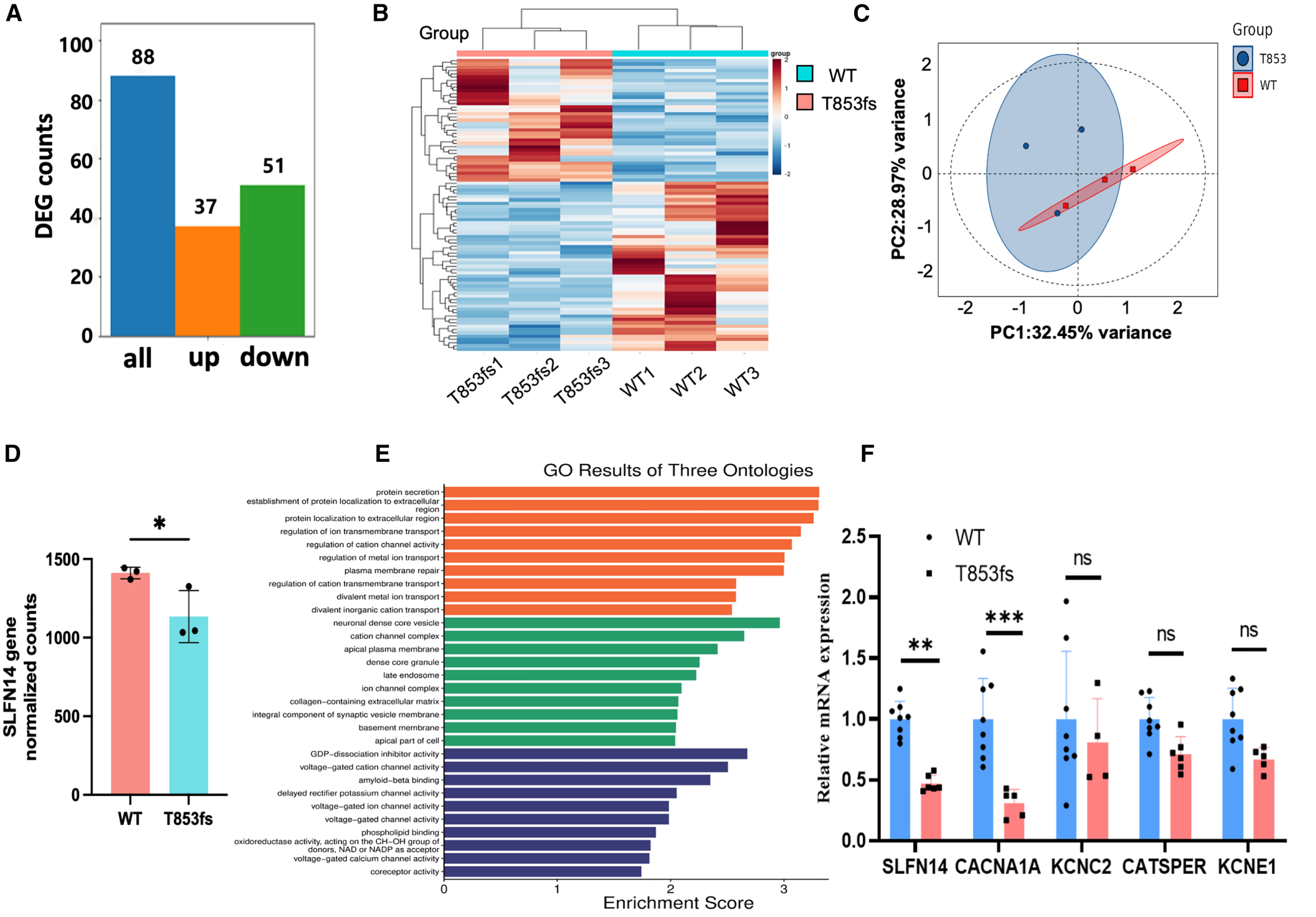

Figure 5. Pathways involved in Thr853fs associated inherited macrothrombocytopenia

(A) Bar plot of counts of up- and down-regulated differentially expressed genes between Meg-01 cells transfected with SLFN14-T853fs and SLFN14-WT. (B) Heatmap showing normalized counts for all differentially expressed genes between WT group and T853fs group. (C) Principal component (PC) analysis plot based on the top 500 genes with the highest variance among samples. (D) Bar plot showing normalized counts for *SLFN14* in Meg-01 cells transfected with SLFN14-T853fs and SLFN14-WT as detected by total RNA-seq. (E) Gene Ontology (GO) analyses of biological functions for differentially expressed genes in WT group and T853fs group. (F) Meg-01 cells were transfected with SLFN14-WT or SLFN14-T853fs overexpression plasmids for 48 h. The expressions of *SLFN14*, *CACNA1A*, *KCNC2*, *CATSPER*, and *KCNE1* were determined by real time qPCR. Data are presented as mean ± SD. Comparisons between two groups were conducted using unpaired two-tailed Student's t test $*p < 0.05$, $**p < 0.05$, $**p < 0.01$, ns = not significant.

the 80S and 60S ribosomal subunits in HEK293T cells, whereas the V220D mutation results in milder rRNA cleavage and less pronounced effects on ribosome-associated mRNA and translational control.[3,21] Interestingly, K208N-deficient mice (the homolog of human K219N) were reported to exhibit elevated red blood cell levels, suggesting additional systemic effects.[13]

In this study, we identified a novel c.2556dupC (T853fs) mutation located in the C-terminal "helicase" domain, distinct from the known hotspot in the AAA domain. This mutation caused a deletion of 27 amino acids in exon 4 of *SLFN14*. Similar to previously reported mutations in the AAA domain, the T853fs variant led to inherited macrothrombocytopenia. *SLFN14* expression was reduced in platelets from the proband and significantly decreased in transfected cells overexpressing the T853fs variant. Consistent with earlier studies, the T853fs variant was associated with fewer dense granules and impaired platelet aggregation in response to ADP stimulation. Additionally, our findings showed that the T853fs mutation disrupted platelet adhesion to fibrinogen (Fg), as evidenced by reduced expression of integrins GPIIb (CD41) and IIIa (CD61), the primary fibrinogen receptors on platelets. These defects collectively contributed to the bleeding tendencies observed in both the proband and her son. No apparent abnormalities were found in granulocytes or erythroid cells. However, unlike mutations in the AAA domain, the T853fs mutation in the helicase domain did not induce ribosomal RNA cleavage.[4]

The C-terminal of SLFN14 contains motifs that align with helicase domains, which are essential for RNA and DNA metabolism. It also contains an ATPase/AAA domain involved in energy-dependent processes, such as protein translation and ribosomal function.[11]

Mutations in *SLFN14* lead to partial misfolding, triggering post-translational degradation and reducing *SLFN14* levels in platelets.[12] It was also reported that *SLFN14* is located in the nucleus because it contains a putative nuclear localization motif in its C-terminus extension.[1,5] Our immunofluorescence assay results showed that the SLFN14 T853fs was located in cytoplasm. This mislocalization is likely due to the truncation of the C-terminal caused by the T853fs mutation, which in turn disturbs the normal function of SLFN14 protein. In animal models, *SLFN14* mutations affect the differentiation of hematopoietic progenitors into erythroid and platelet lineages, suggesting that *SLFN14* may play a broader regulatory role in hematopoiesis beyond megakaryocytes.[22,23]

It was reported that K219N *SLFN14* missense variants cause IT through dysregulation of mTORC1-mediated ribosomal biogenesis, leading to defects in proplatelet formation and mitochondrial organization.[15] Other mutations in the AAA domain significantly upregulate ribosomal protein genes and pathways involved in mitochondrial translation.[13] However, T853fs mutation identified in this study downregulated genes enriched in pathways related to ion channel, ATPase, dense granule, and transmembrane transport. ATP metabolism could affect proplatelet elongation, decrease platelet counts, and weaken platelet aggregation.[24–26] The regulation of ATPase and dense granules was consistent with our observation of reduced dense granules and reduced aggregation in response to ADP in platelets carrying T853fs variants. Calcium signaling is critical for platelet activation, proplatelet formation, and integrin-mediated adhesion.[27,28] Disruption of calcium homeostasis could directly explain the observed defects in platelet function, which is consistent with our results. The distinct mechanistic profile of the T853fs mutation suggests domain-specific therapeutic opportunities. Drugs such as calcium channel activators could possibly restore calcium signaling in patients with helicase domain mutations.

In conclusion, we discovered a novel c.2556dupC (T853fs) mutation in an inherited macrothrombocytopenia with bleeding phenotypes. Different from the previous mutations in the hotspot AAA domain, T853fs mutation in the helicase domain showed no ribosome degradation activity.

MATERIALS AND METHODS

Blood smear

Blood smears were prepared using the Mindray Wright technique, with a control sample from a healthy female volunteer of the same age.

Whole exome sequencing

Following the vendor's recommended protocol, we performed full exon sequencing on the Illumina Novaseq 6000 from LC-Bio Technology Co., Ltd., Hangzhou, China. Mutation hazard levels were classified according to data from the Ensembl Variant Effect Predictor (http://grch37.ensembl.org/info/genome/variation/index.html) and VEP software.

Cell culture and transfection

HEK293T cells were cultured in DMEM with 10% fetal calf serum and 1% pen/strep (both from Biological Industries, Israel). Human megakaryoblast cell line Meg-01 cells were cultured in 1640 medium with 10% fetal calf serum and 1% pen/strep (Biological Industries, Israel). HEK293T cells were plated in 6-well plates at a density of 5×10^5 cells/mL and Meg-01 cells were plated at a density of 1×10^6 cells/mL. After 24 h, cells were transfected using 5-μL Lipofectamine 2000 Transfer Reagent (Thermo Fisher Scientific, USA), 2.5–3 μg plasmids, and 300-μL Opti-MEM (Gibco) per well of a 6-well plate. Subsequent experiments were conducted at least 24 h post-transfection.

Real-time PCR

After 24 h of transfection, total RNA was isolated from HEK293 and Meg-01 cells using the RNA simple Total RNA kit (TIANGEN BIOTECH, China). RNA was converted to cDNA using the TOROIVD QRT Master Mix (TOROIVD, China). Quantitative real-time PCR (real time qPCR) was performed with TB Green Premix Ex Taq II (Tli RNase H Plus) (TAKARA, RR820A, China) and an ABI 7500 real-time fluorescence quantitative PCR machine (Applied Biosystems, USA). GAPDH mRNA was used as an internal reference to calculate the relative expression level of mRNA in each sample.

The primer sequences were as follows.

GAPDH:

Forward: ATGTGTCCGTCGTGGATCTGA

Reverse: ATGCCTGCTTCACCACCTTCTT

SLFN14:

Forward: CCAAAGGCGAAGGGGACT

Reverse: TGAGCAGATGGAGGGGGA

The comparative Ct method was used to quantify mRNA levels. All statistical analyses were performed using GraphPad Prism 8.0.2 (GraphPad Software, San Diego, CA, USA) with the one-way ANOVA test. All experiments were repeated at least three times.

Plasmids construction

All plasmids were purchased from Limibio Biotechnology (China). The sequences of the *SLFN14* primer were as follows.

Forward: 5′-TAATACGACTCACTATAGGG-3′

Reverse: 5′-GCTAGTTATTGCTCAGCGG-3′

All plasmid constructs were verified by sequencing.

Cell/platelet protein extraction

Cells were lysed 48 h after transfection using RIPA lysis buffer (Beyotime, China) according to the manufacturer's instructions. Anticoagulated blood samples were collected with sodium citrate (9 parts blood to 1 part sodium citrate). Total protein was extracted

from the peripheral blood of the patient and healthy donors following the manufacturer's protocol.

Immunoblot analysis and gray value analysis

Total protein concentrations were quantified using a BCA protein assay kit (Beyotime, China) according to the manufacturer's instructions. Protein samples were standardized with 5× loading buffer (Beyotime, China) and boiled at 100°C for 10 min. After SDS-PAGE electrophoresis, proteins were transferred onto polyvinylidene fluoride membranes and blocked in 5% skim milk/TBST buffer for 1 h. Rabbit anti-GAPDH (Proteintech Group, 10494-1-AP, USA) and rabbit anti-SLFN14 (Affinity Biosciences, DF13579-100, USA) antibodies were added, and the membranes were incubated overnight at 4°C. Membranes were washed three times with 1× TBST, then incubated with HRP goat anti-rabbit IgG (H + L) (Proteintech Group, SA00001-2, USA) at room temperature for 1 h. After washing three more times, protein bands were visualized using ECL enhanced chemiluminescence (Shanghai Epizyme Biomedical Technology Co., Ltd.).

ImageJ software was used to quantify and analyze the gray values, normalized by GAPDH. GraphPad Prism 8.0.2 (GraphPad Software) was used to construct relevant graphs using the t test ($\alpha < 0.001$). Values were obtained from three individual experiments.

Cell proliferation assay

After plasmid transfection for 48 h, Meg-01 cells were seeded in 96-well plates at 1×10^4 cells/well in a total volume of 100 μL per well. Each group had three replicate wells and was cultured in a CO_2 incubator. CCK-8 reagent (10μL/well) was added and incubated for 2 h at 37°C. Optical density (OD) values were measured using a microplate reader at 450 nm every 24 h. Data were analyzed and displayed using GraphPad Prism 8.0.2 (GraphPad Software).

Three-dimensional structural analysis of mutant proteins

Human *SLFN14* sequences were downloaded from NCBI. Pymol (https://pymol.org/2/) was used to construct 3D structural maps of the WT and mutant sequences.

Denaturing agarose/formaldehyde gel electrophoresis

A 1.2% denaturing agarose/formaldehyde gel was prepared to load 1.5 μg total RNA of each sample per well. After electrophoresis at 200 V on ice for 25 min in 1× TAE buffer, samples were analyzed using shortwave UV (254 nm).

Platelet aggregation

Anticoagulated blood was collected with sodium citrate (10 mL) and centrifuged at 200 g at room temperature for 10 min to obtain platelet-rich plasma (PRP). The remaining blood was centrifuged at 2000 g at room temperature for 10 min to obtain platelet-poor plasma (PPP). PPP was used to adjust the platelet count in PRP to 1×10^8/L. ADP (10 μM) and AA (1 mM) were added to PRP (10 μL per well). OD values were measured using a microplate reader at 490 nm after 0–15 min of oscillation. Platelet aggregation rate was calculated as ((1 − OD value before adding agonist)/OD value after adding agonist) × 100%. Each group had three replicate wells. Data were analyzed using GraphPad Prism 8.0.2 (GraphPad Software).

Platelet spreading

Coverslips were prepared according to Fletcher's description.[1] Fourteen-millimeter coverslips were coated with 100 μg/mL fibrinogen (Shandong Taibang Biological Products Co., Ltd., China) overnight at 4°C and then blocked with 5 mg/mL sterile BSA at room temperature for 1 h. After washing in PBS three times, 300 μL of 2×10^7/mL platelets were added per well and incubated for 45 min at 37°C. Unattached platelets were removed, washed with PBS, and fixed with 4% paraformaldehyde at room temperature for 5 min. After washing in PBS, platelets were permeabilized with 0.5% Triton X-100 for 10 min and stained with Alexa Fluor 488-conjugated phalloidin. A fluorescence microscope was used to take photos, and ImageJ software was used to analyze images. GraphPad Prism 8.0.2 (GraphPad Software) was used to construct relevant graphs using the t test ($\alpha < 0.001$). Values were obtained from three individual experiments.

Transmission electron microscope

Fresh anticoagulated blood with sodium citrate was centrifuged at 2000 g for 10 min. The upper serum layer was removed, and 2.5% glutaraldehyde was added dropwise and gently along the wall. The sample was centrifuged at 2000 g again for 10 min and allowed to sit for 30 min to ensure complete penetration by the glutaraldehyde. A needle was used to peel off the upper layer of platelets, which were transferred to new 2.5% glutaraldehyde and stored at 4°C for later experiments. Pretreated samples were washed in PBS three times and then post-fixed with 1% osmium acid at room temperature in the dark. Samples were rinsed in distilled water and stained with uranyl acetate solution at room temperature in the dark for 1 h. After gradient dehydration in acetone, samples were embedded, sliced, and stained with lead citrate before photographing.

Granules in platelets were counted, and the average diameter of each platelet (average of long and short axes) was measured. Fifteen platelets per group were counted. GraphPad Prism 8.0.2 (GraphPad Software) was used to analyze data using the t test ($\alpha < 0.001$).

RNA-seq analysis

Total RNA from WT-SLFN14 and T853fs-SLFN14 plasmid-transfected Meg-01 cells was individually extracted using Trizol (Invitrogen). mRNA sequencing was performed by Personalbio using Illumina HiSeq. Mapping was conducted with HISAT2. GO enrichment analysis was applied to compare platelet-associated genes between the WT and T853fs variants.

DATA AND CODE AVAILABILITY

All of the data that support the findings of this study are available from the corresponding author upon reasonable request.

ACKNOWLEDGMENTS

This work was supported by the National Natural Science Foundation of China (82272784 to L. Zhu), the Wenzhou Science and Technology Bureau (Y20220126 to H.X.), Beijing Natural Science foundation (7252006 to L. Zhu), and the Zhejiang Province Science and Technology Plan Research and Xinmiao Talent Program (grant no. 2023R413093).

AUTHOR CONTRIBUTIONS

L. Zhu, H.X., C.L., and R.O. designed the research. H.X., S.T., J.S., M.Y., and X.Y. conducted the experiments. L. Zhang, S.T., J.S., and X.Y. participated in the study design. L. Zhu and S.T. analyzed the data and wrote the manuscript. M.Z. and H.T. contributed to the bioinformatics analyses. L.B. contributed to the scientific supervision. H.X. performed additional experiments requested during the revision process and assisted in manuscript revision and language editing. All authors read and approved the final manuscript.

DECLARATION OF INTERESTS

The authors declare no competing interests.

SUPPLEMENTAL INFORMATION

Supplemental information can be found online at https://doi.org/10.1016/j.omtn.2025.102554.

REFERENCES

1. Fletcher, S.J., Johnson, B., Lowe, G.C., Bem, D., Drake, S., Lordkipanidzé, M., Guiú, I.S., Dawood, B., Rivera, J., Simpson, M.A., et al. (2015). SLFN14 mutations underlie thrombocytopenia with excessive bleeding and platelet secretion defects. J. Clin. Investig. *125*, 3600–3605.
2. Marconi, C., Di Buduo, C.A., Barozzi, S., Palombo, F., Pardini, S., Zaninetti, C., Pippucci, T., Noris, P., Balduini, A., Seri, M., and Pecci, A. (2016). SLFN14-related thrombocytopenia: identification within a large series of patients with inherited thrombocytopenia. Thromb. Haemost. *115*, 1076–1079.
3. Andrew, T. (2015). Characterization of Novel Ribosome-Associated Endoribonuclease SLFN14 from Rabbit Reticulocytes. Biochemistry *54*, 3286–3301.
4. Polokhov, D., Fedorova, D., Ignatova, A., Ponomarenko, E., Rashevskaya, E., Martyanov, A., Podoplelova, N., Aleksenko, M., Mersiyanova, I., Seregina, E., et al. (2023). Novel SLFN14 mutation associated with macrothrombocytopenia in a patient with severe haemorrhagic syndrome. Orphanet J. Rare Dis. *18*, 74.
5. Jo, U., and Pommier, Y. (2022). Structural, molecular, and functional insights into Schlafen proteins. Exp. Mol. Med. *54*, 730–738.
6. Hou, P., Hao, W., Qin, B., Li, M., Zhao, R., and Cui, S. (2023). Structural and biochemical characterization of Schlafen11 N-terminal domain. Nucleic Acids Res. *51*, 7053–7070.
7. Metzner, F.J., Huber, E., Hopfner, K.P., and Lammens, K. (2022). Structural and biochemical characterization of human Schlafen 5. Nucleic Acids Res. *50*, 1147–1161.
8. Liu, F., Zhou, P., Wang, Q., Zhang, M., and Li, D. (2018). The Schlafen family: complex roles in different cell types and virus replication. Cell Biol. Int. *42*, 2–8.
9. Qi, F., Alvi, E., Ogawa, M., Kobayashi, J., Mu, A., and Takata, M. (2023). The ribonuclease domain function is dispensable for SLFN11 to mediate cell fate decision during replication stress response. Genes Cells *28*, 663–673.
10. Komori, K., Hidaka, M., Horiuchi, T., Fujikane, R., Shinagawa, H., and Ishino, Y. (2004). Cooperation of the N-terminal Helicase and C-terminal Endonuclease Activities of Archaeal Hef Protein in Processing Stalled Replication Forks. J. Biol. Chem. *279*, 53175–53185.
11. Stapley, R.J., Pisareva, V.P., Pisarev, A.V., and Morgan, N.V. (2020). SLFN14 gene mutations associated with bleeding. Platelets *31*, 407–410.
12. Fletcher, S.J., Pisareva, V.P., Khan, A.O., Tcherepanov, A., Morgan, N.V., and Pisarev, A.V. (2018). Role of the novel endoribonuclease SLFN14 and its disease-causing mutations in ribosomal degradation. RNA *24*, 939–949.
13. Stapley, R.J., Smith, C.W., Haining, E.J., Bacon, A., Lax, S., Pisareva, V.P., Pisarev, A. V., Watson, S.P., Khan, A.O., and Morgan, N.V. (2021). Heterozygous mutation SLFN14 K208N in mice mediates species-specific differences in platelet and erythroid lineage commitment. Blood Adv. *5*, 377–390.
14. Scheiner, B., Kirstein, M.M., Popp, S., Hucke, F., Bota, S., Rohr-Udilova, N., Reiberger, T., Müller, C., Trauner, M., Peck-Radosavljevic, M., et al. (2018). Association of platelet count and mean platelet volume with overall survival in patients with cirrhosis and unresectable hepatocellular carcinoma. Z. Gastroenterol. *56*, e50.
15. Ver Donck, F., Ramaekers, K., Thys, C., Van Laer, C., Peerlinck, K., Van Geet, C., Eto, K., Labarque, V., and Freson, K. (2023). Ribosome dysfunction underlies SLFN14-related thrombocytopenia. Blood *141*, 2261–2274.
16. Armstrong, P.C., and Peter, K. (2012). GPIIb/IIIa inhibitors: from bench to bedside and back to bench again. Thromb. Haemost. *107*, 808–814.
17. Kammers, K., Taub, M.A., Rodriguez, B., Yanek, L.R., Ruczinski, I., Martin, J., Kanchan, K., Battle, A., Cheng, L., Wang, Z.Z., et al. (2021). Transcriptional profile of platelets and iPSC-derived megakaryocytes from whole-genome and RNA sequencing. Blood *137*, 959–968.
18. Warren, J.T., and Di Paola, J. (2022). Genetics of inherited thrombocytopenias. Blood *139*, 3264–3277.
19. Pape, T., Wintermeyer, W., and Rodnina, M.V. (2000). Conformational switch in the decoding region of 16S rRNA during aminoacyl-tRNA selection on the ribosome. Nat. Struct. Biol. *7*, 104–107.
20. Snider, J., Thibault, G., and Houry, W.A. (2008). The AAA+ superfamily of functionally diverse proteins. Genome Biol. *9*, 216.
21. Neubauer, C., Gao, Y.G., Andersen, K.R., Dunham, C.M., Kelley, A.C., Hentschel, J., Gerdes, K., Ramakrishnan, V., and Brodersen, D.E. (2009). The Structural Basis for mRNA Recognition and Cleavage by the Ribosome-Dependent Endonuclease RelE. Cell *139*, 1084–1095.
22. Psaila, B., and Mead, A.J. (2019). Single-cell approaches reveal novel cellular pathways for megakaryocyte and erythroid differentiation. Blood *133*, 1427–1435.
23. Meinders, M., Hoogenboezem, M., Scheenstra, M.R., De Cuyper, I.M., Papadopoulos, P., Németh, T., Mócsai, A., van den Berg, T.K., Kuijpers, T.W., and Gutiérrez, L. (2016). Repercussion of Megakaryocyte-Specific Gata1 Loss on Megakaryopoiesis and the Hematopoietic Precursor Compartment. PLoS One *11*, e0154342.
24. Patel, S.R., Richardson, J.L., Schulze, H., Kahle, E., Galjart, N., Drabek, K., Shivdasani, R.A., Hartwig, J.H., and Italiano, J.E. (2005). Differential roles of microtubule assembly and sliding in proplatelet formation by megakaryocytes. Blood *106*, 4076–4085.
25. Marelli-Berg, F.M., and Jangani, M. (2018). Metabolic regulation of leukocyte motility and migration. J. Leukoc. Biol. *104*, 285–293.
26. Baracca, A., Barogi, S., Carelli, V., Lenaz, G., and Solaini, G. (2000). Catalytic Activities of Mitochondrial ATP Synthase in Patients with Mitochondrial DNA T8993G Mutation in the ATPase 6 Gene Encoding Subunit a. J. Biol. Chem. *275*, 4177–4182.
27. Johnson, G.J., Leis, L.A., and Francis, G.S. (1986). Disparate effects of the calcium-channel blockers, nifedipine and verapamil, on alpha 2-adrenergic receptors and thromboxane A2-induced aggregation of human platelets. Circulation *73*, 847–854.
28. Kazandzhieva, K., Mammadova-Bach, E., Dietrich, A., Gudermann, T., and Braun, A. (2022). TRP channel function in platelets and megakaryocytes: basic mechanisms and pathophysiological impact. Pharmacol. Ther. *237*, 108164.

OMTN, Volume *36*

Supplemental information

Novel mutation *SLFN14* T853fs associated with inherited macrothrombocytopenia

Haixiao Xie, Shiyi Tang, Jianmin Shao, Ming Yang, Huida Tong, Linhua Zhang, Mingzhu Zhong, Xiaomin Yu, Laixi Bi, Yuming Wang, Rongying Ou, Chen Ling, and Liqing Zhu

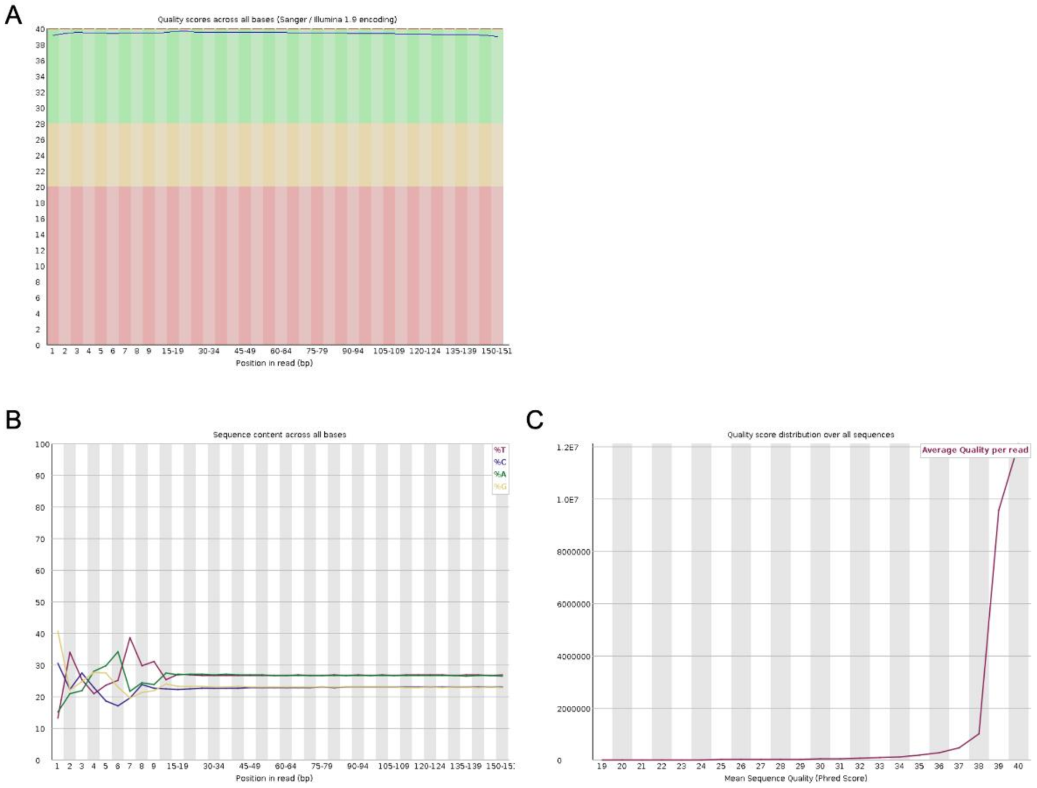

Figure S1. Representive quality control analysis of RNA-seq. (A) Per-base sequence quality scores (y-axis) across read positions (x-axis in bp). Colors (green [high] to red [low]) indicate quality variations. **(B)** Per-base sequence composition. Nucleotide content percentage (y-axis) across read positions (x-axis in bp). Lines represent %T (red), %C (blue), %A (green) and %G (yellow). **(C)** Read quality distribution. Y-axis: read count. X-axis: mean sequence quality.
